# Prospective assessment of diagnostic efficacy and safety of Sonazoid^TM^ and SonoVue^®^ ultrasound contrast agents in patients with focal liver lesions

**DOI:** 10.1007/s00261-021-03010-1

**Published:** 2021-06-16

**Authors:** Ke Lv, Hongyan Zhai, Yuxin Jiang, Ping Liang, Hui-Xiong Xu, Lianfang Du, Yi-Hong Chou, Xiaoyan Xie, YuKun Luo, Young Joon Lee, Jae Young Lee, Bing Hu, Baoming Luo, Yi Wang, Ying Luan, Christina Kalli, Kun Chen, Wenping Wang, Ja-Der Liang

**Affiliations:** 1 Department of Ultrasound, Peking Union Medical College Hospital, Peking Union Medical College, Chinese Academy of Medical Sciences, Beijing, 100730 China; 2 Chinese PLA General Hospital, Beijing, China; 3 Shanghai Tenth People’s Hospital, Tongji University, Shanghai, China; 4 Shanghai First People’s Hospital, Shanghai, China; 5 Taipei Veterans General Hospital, Taipei, China; 6 The First Affiliated Hospital of Sun-Yatsen University, Guangzhou, China; 7 Seoul St. Mary’s Hospital, Seoul, South Korea; 8 Seoul National University Hospital, Seoul, South Korea; 9 Shanghai Sixth Hospital, Shanghai, China; 10 Sun Yet-Sen Memorial Hospital, Sun Yet-Sen University, Guangzhou, China; 11 Shanghai Hua Shan Hospital, Fudan University, Shanghai, China; 12 GE HealthcareLife Sciences, Piscataway, USA; 13 Zhongshan Hospital Fudan University, Shanghai, China; 14 Tianjin Medical University General Hospital, Tianjin, China; 15 National Taiwan University Hospital, Taipei, China

**Keywords:** Contrast-enhanced ultrasound, Focal liver lesions, Characterization, Vascular imaging, Diagnostic accuracy

## Abstract

**Objectives:**

To assess the respective diagnostic value of Sonazoid™ and SonoVue® for characterizing FLLs as benign or malignant and the corresponding safety.

**Methods:**

This prospective Phase 3 study was conducted at 17 centres in China and Korea (May 2014 to April 2015); 424 patients (20 to 80 years) with at least 1 untreated focal liver lesion (FLL) (< 10 cm in diameter) underwent a contrast-enhanced ultrasound (CEUS) examination (218 received Sonazoid of 0.12 μL microbubbles/kg; 206 received SonoVue of 2.4 mL). Three independent blinded readers evaluated pre- and post-contrast images characterising the FLLs as benign or malignant.

**Results:**

Sonazoid-enhanced and SonoVue-enhanced ultrasound provided a statistically significant improvement in specificity for all 3 readers comparing to unenhanced ultrasound (for Sonazoid: *p* = 0.0093, < 0.0001, 0.0011; for SonoVue: *p* = 0.002, 0.03, 0.12, respectively). Difference in accuracy improvement between the 2 groups was within the pre-specified non-inferiority margin of 20% for all 3 readers (6.1%, 95% CI: − 5.0 to 17.2; − 7.5%, 95% CI: − 18.4 to 3.5; − 0.3%, 95% CI: − 11.3 to 10.7).

The diagnostic confidence level for all 3 readers increased with post-contrast images relative to pre-contrast images. Both contrast agents were well tolerated.

**Conclusion:**

Results showed a similar efficacy for Sonazoid™ and SonoVue® in diagnosing FLLs as benign or malignant, and underlined the benefit of CEUS imaging over unenhanced ultrasound imaging in reaching a confident diagnosis without having to refer patients for additional imaging exams.

## Introduction

Contrast-enhanced ultrasound (CEUS) has an important role in diagnostic strategies of focal liver lesions (FLLs) as underlined in the WFUMB-EFSUMB guidelines [1]. Comparing to conventional B-mode and colour Doppler ultrasound imaging, CEUS has higher efficacy in detection and characterization of FLLs [2, 3]. Moreover, CEUS has demonstrated similar diagnostic performance to computed tomography (CT) and magnetic resonance imaging (MRI), with advantages including real-time scanning and no radiation exposure [4–7].

The ultrasound contrast agents (UCA) currently used in CEUS are gas-filled microbubbles (3–10 μm diameter) stabilized by a flexible shell such as phospholipids or albumin. These microbubbles act as resonant entities in an ultrasound field, generating nonlinear scattered signals to discriminate the blood flow from surrounding tissue [8, 9]. The UCAs used in this study are Sonazoid™,^1^ (perfluorobutane microbubbles, GE Healthcare, Oslo, Norway), and SonoVue®,^2^ (sulphur hexafluoride microbubbles, Bracco, Milan, Italy). Sonazoid consists of perfluorobutane (C_4_F_10_) gas stabilized by a monomolecular membrane of hydrogenated egg phosphatidylserine; SonoVue is composed of sulfurhexfluoride gas encapsulated by a phospholipid shell. Both agents have been used for more than a decade in clinical applications for CEUS of FLLs, leading to their increased use in routine practice across the world [6, 10–15].

During CEUS examinations, FLLs can be assessed after intravenous injection of microbubbles through continuous real-time observation of contrast enhancement dynamics of the tumour vasculature and the surrounding liver parenchyma for up to 4–6 min post-injection (vascular phase) [7, 16]. The specific Kupffer cells uptake of Sonazoid has been reported to be related to the phagocytosis of the contrast agent microbubbles by liver specific macrophages which leads to the capacity to image patients over an extended late phase (named “Kupffer phase” or “post-vascular phase”) [17, 18]. The contrast enhancement patterns of vascular and Kupffer phase can enable the characterization of FLLs and the detection or rule-out the presence of lesions [1, 3, 17–19]. Kang et al. [20] compared the SonoVue and Sonazoid for the Diagnosis of Hepatocellular Carcinoma in Individuals with High Risk and made the conclusion that noninvasive US diagnosis of hepatocellular carcinoma using perfluorobutane-enhanced US had higher diagnostic performance than sulfur hexafluoride-enhanced US, without loss of specificity.

The objective of the present study was to assess the respective diagnostic value of Sonazoid and SonoVue for characterizing FLLs as benign or malignant and their corresponding safety. To the best of our knowledge, this is the first multicenter prospective study comparing the two ultrasound contrast agents.

## Material and methods

This Phase 3 prospective study was approved by an Independent Ethics Committee or Independent Review Board at each clinical site according to national or local regulations with informed consent.

### Patient

Enrolled subjects must have had at least one untreated FLL that can be visualized by non-contrast-enhanced ultrasound but less or equal to eight lesions (excluding cysts) smaller than 10 cm in diameter. The patient must have had a dynamic contrast-enhanced CT (CE-CT) or contrast-enhanced MRI (CE-MRI) examination (as the reference examination) within the past month or was scheduled to have one in the month following inclusion in the study. If a subject had multiple FLLs, the most suitable lesion was selected as the target lesions. The most suitable lesion refers to the size of the lesion is between 1 and 8 cm, two-dimensional ultrasound can clearly show the lesion, and the lesion is a solid lesion, not a cyst. The target lesion might receive biopsy and histopathological examination was performed (i.e., for cases histopathology would serve as the reference diagnosis in vascular phase). Where comparative imaging (CE-CT and/or CE-MRI) was a component of the reference diagnosis, the lesion selected had or was to have a formal diagnosis based on a combination of imaging and clinical data/medical history.

Exclusion criteria were ongoing chemotherapy or radiation therapy; allergies to eggs or egg products (hydrogenated egg phosphatidylserine sodium in Sonazoid may cause allergic symptoms); hypersensitivity to sulphur hexafluoride or any other component of SonoVue; administration or scheduled administration of another contrast agent within 24 h before or after study participation; thrombosis within the liver, portal, or mesenteric veins. In addition, the following cardiac and pulmonary contra-indications for the class of ultrasound contrast agents were considered: recent acute coronary syndrome or clinically unstable ischaemic cardiac disease; adult respiratory distress syndrome, severe emphysema, pulmonary vasculitis, or history of pulmonary emboli; known right-to-left shunt, severe pulmonary hypertension or uncontrolled systemic hypertension.

Assuming the accuracy improvement was 40% for both SonoVue and Sonazoid, with a non-inferiority margin of 20%, a sample size of 96 subjects per group (i.e., 192 subjects in total) was required to ensure 80% power and a 5% type I error rate. The minimum sample size for the trial countries were 140 subjects per group in mainland China and Taiwan, and 15 per group in Korea, resulting in 310 subjects in total. During 11 months, a total of 424 subjects (218 received Sonazoid and 206 received SonoVue) were enrolled at 17 centres in China and Korea. All subjects were included in safety evaluation. As specified in the protocol, patients who did not have reference diagnosis within one month prior and following inclusion in the study (*n* = 16) were excluded from the efficacy analysis. Additionally, before the enrolment of the main study subjects, site training was performed with 70 patients fulfilling the same inclusion and exclusion criteria, to help investigators become familiar with the use of Sonazoid or SonoVue. This led to 338 patients (169 each in the Sonazoid and SonoVue groups) to be included in the efficacy evaluation ultimately.

All lesions were divided into six categories: Hepatocellular Carcinoma (HCC), liver metastasis, other malignant lesions, hemangioma, focal nodular hyperplasia (FNH) and other benign lesions. Other benign lesions included focal nodular hyperplasia, inflammatory pseudotumor, low lipid areas of heterogeneous fatty liver, atypical hemangiomas and Hepatic inflammatory lesion; other malignant lesions included bile duct cell carcinoma and other lesions with signs of malignancy. The diagnostic criteria of CEUS were as follows: for patients with liver cirrhosis, the presence of rich blood supply in arterial phase with washout in late phase was the typical enhancement pattern of HCC. For the metastatic lesion, the rim-like enhancement at the tumor periphery with the black hole in delayed phase was the typical pattern. For hemangioma, the diagnostic enhancement pattern was globular-like pooling in the periphery with hypoperfusion in tumors, which continued till the late phase. For FNH, the presence of a spoke-wheel pattern in the early vascular phase with hyperenhancement in the late vascular phase was the most typical pattern [21].

### Randomization

Subjects would be randomly assigned to receive Sonazoid or SonoVue in a 1:1 ratio. The randomization scheme was generated by an independent statistician different from the study team. The allocation sequence was generated via central randomization with a block size of 4. Every site had been assigned a fixed number of randomly allocated treatment codes which far exceeded the maximum number of subjects allowable per site and were associated with the subject IDs in a sequential order. The subjects enrolled in the study was assigned with the subject ID in a sequential order.

The randomization scheme was provided to sponsor IMP dispense group by the unblinded independent statistician who generated the scheme. The sponsor IMP dispense group then printed the subject ID and batch number on each vial label and shipped the kit to the sites.

Because optimal imaging with Sonazoid and SonoVue required different ultra sound machine settings, the ultrasound operator could not be blinded with respect to which contrast agent was injected. One or more members of the study staff at each site would be responsible for preparing the prescribed IMP for each subject in accordance with the randomization schedule. The unblinded person who prepared and injected the IMP could not participate in any safety monitoring or allow team members who performed safety assessments to see which IMP had been prepared and injected.

Subjects received Sonazoid or SonoVue under the direct supervision of study personnel at the study sites and in compliance with the randomisation schedule generated by Winfield Consulting (PO Box 5497, Buffalo Grove, IL 60089, USA). Each administration volume was checked and the vial number, subject number, and injection volume per administration were recorded in each subject’s CRF.

### Ultrasound contrast agents

Subjects were randomized to receive a single intravenous dose of either Sonazoid (0.12 µL/kg of perflubutane microbubbles) or SonoVue (2.4 mL) in a 1:1 ratio according to a priori computer-generated list. Sonazoid and SonoVue were reconstituted in accordance to the manufacturers’ instructions. The injection of both contrast agents was followed by a flush with 5–10 mL of 0.9% Sodium Chloride at a recommended injection rate of approximately 1 mL/s.

### Ultrasound

All ultrasound scanners used in the study (GE LOGIQ™ E9,GE Healthcare, Milwaukee, USA; Philips iU22™, Philips Healthcare, Bothell, USA; and Toshiba Aplio™ 500, Canon Medical Systems, Zoetemeer, the Netherlands) were equipped with low frequency curved-array transducers for abdominal exams. Ultrasound settings for the pre-contrast and post-contrast liver examinations were predefined as a starting point and were adjusted to optimise the image quality for each patient by the investigators. Part of the pre-sets (mechanical index (MI), frequency, frame rate, depth and focus) are displayed in Table 1 [18–20]. Digital video files and still images were recorded in the Digital Imaging and Communications in Medicine (DICOM) format.

**Table 1:** Pre-defined settings for study ultrasound scanners

Ultrasound scanner	Mechanical Index (MI)		Frequency	Frame rate	Depth	Focus
	Sonazoid	SonoVue				
GE LOGIQ E9	0.15–0.31	0.06–0.2	(Gen), (Res) or adjust according to patient	9–11 Hz	Adjust according to lesion depth	Place focus at the bottom of the lesion, and ideally at the bottom of the field of view
Philips iU22	0.18–0.19	0.06–0.07	Gen/Pen/Res/HRes/CGen/CRes/CPen (Or patient dependent)	9–10 Hz		
Toshiba Aplio 500	0.15–0.18	0.07–0.08	3.0 h, 3.5 h	5 Hz		

Due to the lack of standardization in calculating MI, the scanners have different optimal MI settings

### Pre-contrast imaging

Fundamental or tissue harmonic B-mode imaging was performed in sagittal and transverse planes to image the target FLL and the whole liver (10–30 s sweeps). Colour Doppler modes were used to characterise the vasculature in and around the target lesion.

### Post-contrast imaging

Post-contrast imaging was performed immediately after the dose administration in contrast-specific ultrasound mode using the same field of view as for the pre-contrast imaging. The dual-screen view that displayed the contrast-enhanced image and the fundamental B-mode image were utilized. For CEUS with Sonazoid, continuous scanning of the target lesion was recorded from the beginning of injection to 2 min after injection (vascular phase imaging). The subsequent scans at 3 min, 4 min, 5 min and 10 min were intermittent acquisitions, respectively. 10 min after injection of contrast media (in the kupffer phase), the whole liver was scanned following the same procedures as those for unenhanced ultrasound. For SonoVue, the target lesion was also imaged from the beginning of injection to 2 min after injection. 3 min, 4 min, 5 min images of the target lesion were scanned and recorded. The difference was that the whole liver scanning was performed 5 min (in the late phase) after injection and at 10 min the target lesion was scanned again. Second injection was not allowed in all lesions.

### Reference diagnosis

The reference diagnosis for the target FLL for each patient were provided by onsite investigators based on the results of dynamic CE-CT or CE-MRI examination, or histopathology based on biopsy or surgery if available, with the CEUS exam result excluded. The target FLLs were identified and cross-matched on both the ultrasound and the reference imaging exam based on the lesion size, shape and location by trained ultrasound, CT and MRI technologists at the study sites.

### Blinded image evaluation

All images were blinded and randomised and read offsite by 3 independent physicians with at least 5 years of CEUS experience. The blinded readers evaluated the images without clinical information or knowledge of the reference diagnosis. The imaging data from each patient were divided into two data sets, i.e., pre-contrast images and post-contrast images. Pre- and post-contrast images were randomized separately (i.e., assigned with different sets of randomized codes) and assessed in two separate reading sessions by characterizing the target FLL as benign versus malignant lesions, and the confidence of diagnosis was rated.

The blinded readers evaluated the pre-contrast images in accordance to standard clinical practice and based their diagnosis on aspects of lesion features, appearance of surrounding liver and lesion-related vascularity patterns. If the FLL had anechoic halo around, hada sense of occupation or with high resistance arterial blood flow in conventional ultrasound, the lesion could be classified as malignant. CEUS characterization of the liver lesions was done by assessing the enhancement patterns in the different phases of vascular imaging (arterial phase, portal-venous phase, late, and very late phase) in accordance with WFUMB-EFSUMB guidelines [1]. For patients with insufficient CEUS imaging, if the blinded readers gave the same conclusion, the conclusion would be the diagnosis of the focal liver lesion. If there were discrepancies, as long as two of them give the same conclusion, it would be adopted as a CEUS diagnosis.

### Safety evaluation

Safety variables were assessed by on-site investigators or staff who were blinded to study contrast agents at baseline before contrast injection, at approximately 4 h post-injection, and at nominally 24 h and 72 h via phone call. All adverse events (AEs), serious AEs, changes in vital signs, clinical laboratory variables and physical examination status, and injection site monitoring were recorded and assessed.

### Statistical analyses

A statistical software package (SAS version 9.2, SAS Analytics, Marlow, UK) was used for the statistical analysis. The significance level was 0.05 for two-sided tests and 0.025 for one-sided tests. Efficacy analyses were performed using the efficacy population including subjects who received either Sonazoid or SonoVue, with pre-contrast and post-contrast images recorded, and a reference standard examination performed.

The primary objective of the study was to demonstrate non-inferiority of Sonazoid compared with SonoVue with respect to the diagnostic accuracy improvement from unenhanced to enhanced ultrasound examination (the percentage of cases whose blinded reader’s diagnosis agreed with the reference diagnosis for post-contrast imaging, but not for pre-contrast imaging). For each reader, the difference and exact 95% binomial confidence interval (CI) of the accuracy improvement between Sonazoid and SonoVue were calculated. The fixed margin method outlined in the guidelines of the FDA was used to determine the 20% non-inferiority margin [22].

For the analysis, the improvement from pre- to post-contrast images in sensitivity, specificity, and overall agreement (OA), respectively, defined as % patients whose blinded reader diagnosis agrees with reference standards were tested by McNemar’s test. The increase in area under the receiver operating characteristic curves (AUC) was estimated using Mann–Whitney statistics. Both the intra-reader and inter-reader viabilities were assessed by the percent agreement and the kappa statistics.

## Results

### Efficacy results

#### Characterisation

**Fig. 1:**
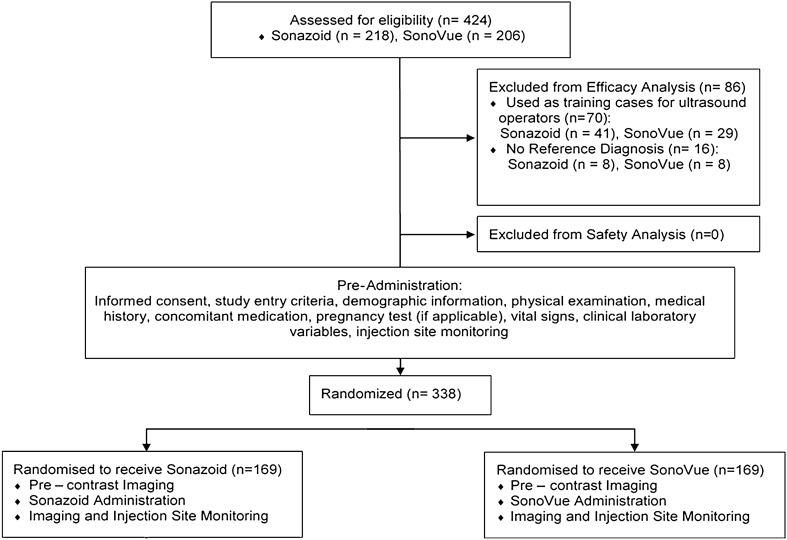
The flow diagram of the study

Table 2 shows demographics and baseline characteristics of the study population. There were no significant differences between the Sonazoid and SonoVue groups for any of the variables (*p* > 0.05). Note that 227 patients (54%) had a history of liver disease, with hepatitis B and primary or metastatic cancer being most commonly reported. The distribution of reference diagnoses for the target FLLs was similar across the Sonazoid and SonoVue groups as summarised in Table 2 (Fig. 1).

**Table 2:** Demographics and characteristics of the study population

Variable	Sonazoid group *N* = 218	SonoVue group *N* = 206	*p*-value^a^
Age, mean ± SD	54.5 ± 12.6	52.2 ± 13.8	0.07
Gender, *n* (%)			
Male	137 (52.3%)	125 (47.7%)	0.65
Female	81 (50.0%)	81 (50.0%)	–
Body Mass Index, mean ± SD (BMI, kg/m^2^)	24.1 ± 3.5	23.8 ± 3.4	0.20
Any liver disease history, *n* (%)	119 (54.6%)	108 (52.4%)	0.66
Hepatitis B	36 (16.5%)	30 (14.6%)	–
Hepatitis C	3 (1.4%)	4 (1.9%)	–
Primary or metastatic cancer	34 (15.6%)	27 (13.1%)	–
Benign disease	20 (9.2%)	15 (7.3%)	–
Cirrhosis	7 (3.2%)	2 (1.0%)	–
Other	19 (8.7%)	30 (14.6)	–
Efficacy population, *n*	169	169	–
Target lesion reference diagnosis^b^, n (%)			–
Malignant			
Hepatocellular carcinoma (HCC)	65 (38%)	61 (36%)	0.28
Metastasis	15 (9%)	11 (7%)	–
Other	4 (2%)	2 (1%)	–
Benign			–
Focal nodular hyperplasia (FNH)	13 (8%)	15 (9%)	–
Haemangioma	53 (31%)	61 (36%)	–
Other	19 (11%)	19 (11%)	–

Some percentages do not add up to 100 due to rounding errors

*SD* standard deviation

^a^The *p*-values are based on two-sample *t*-test for continuous variables and based on Chi-square test for categorical variables

^b^Percentages are based on number of patients in the efficacy population. The *p*-value is based on the comparison of dichotomized reference diagnosis (malignant vs benign) between the two groups

**Fig. 2:**
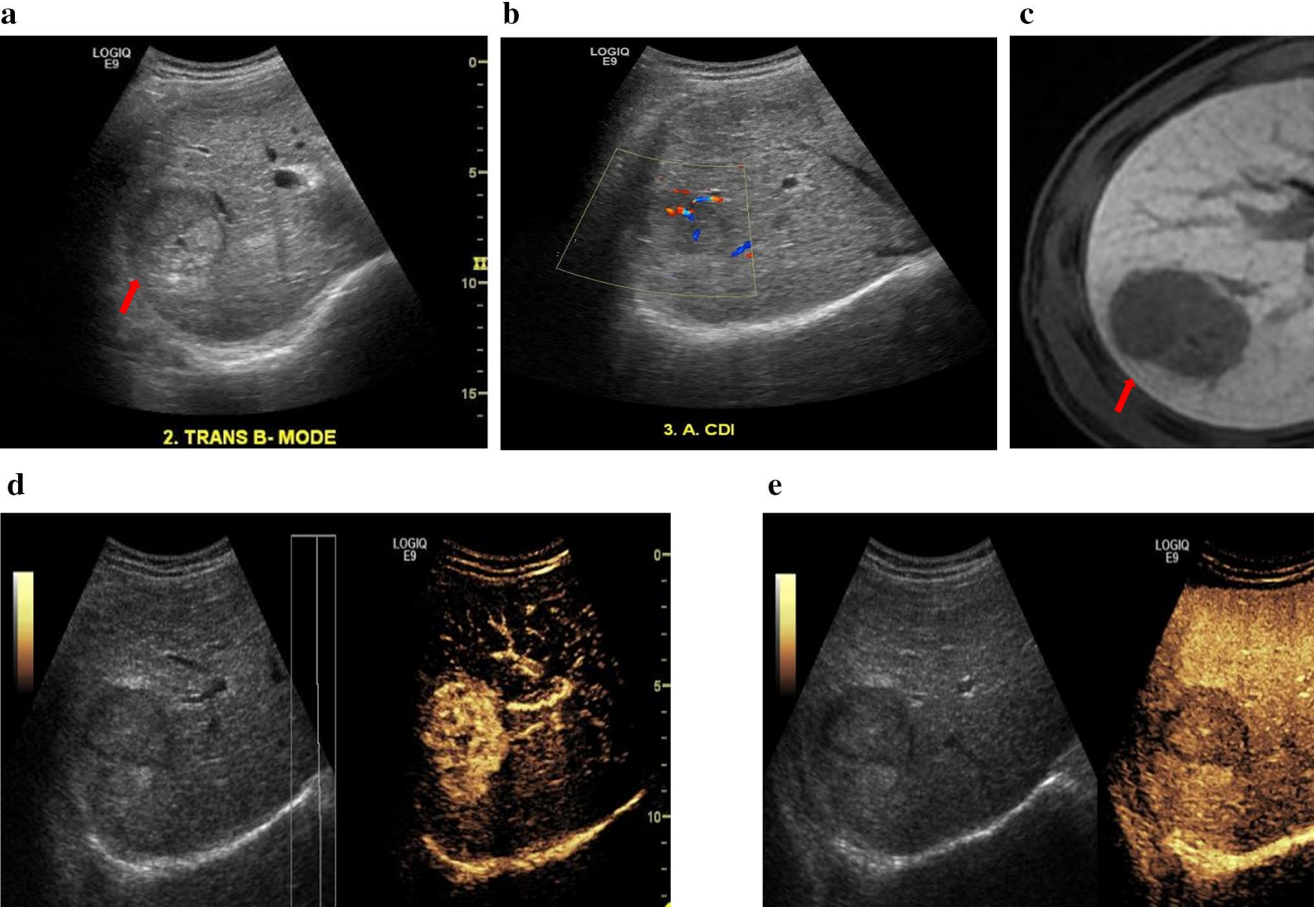
51-year-old male patient with hepatocellular carcinoma (HCC). **a** Pre-contrast harmonic B-mode shows isoechoic HCC. **b** In Colour Doppler imaging HCC shows some peripheral and central vascularity. **c** CE-MRI used as the reference diagnosis, indicating the HCC in the hepatobiliary phase. **d** Arterial phase post-Sonazoid injection shows homogeneous hyper-enhancement. **e** Kupffer phase at approximately 10 min shows washout with well-defined lesion margins, hypo-enhancement and strong contrast retention in the liver parenchyma

**Fig. 3:**
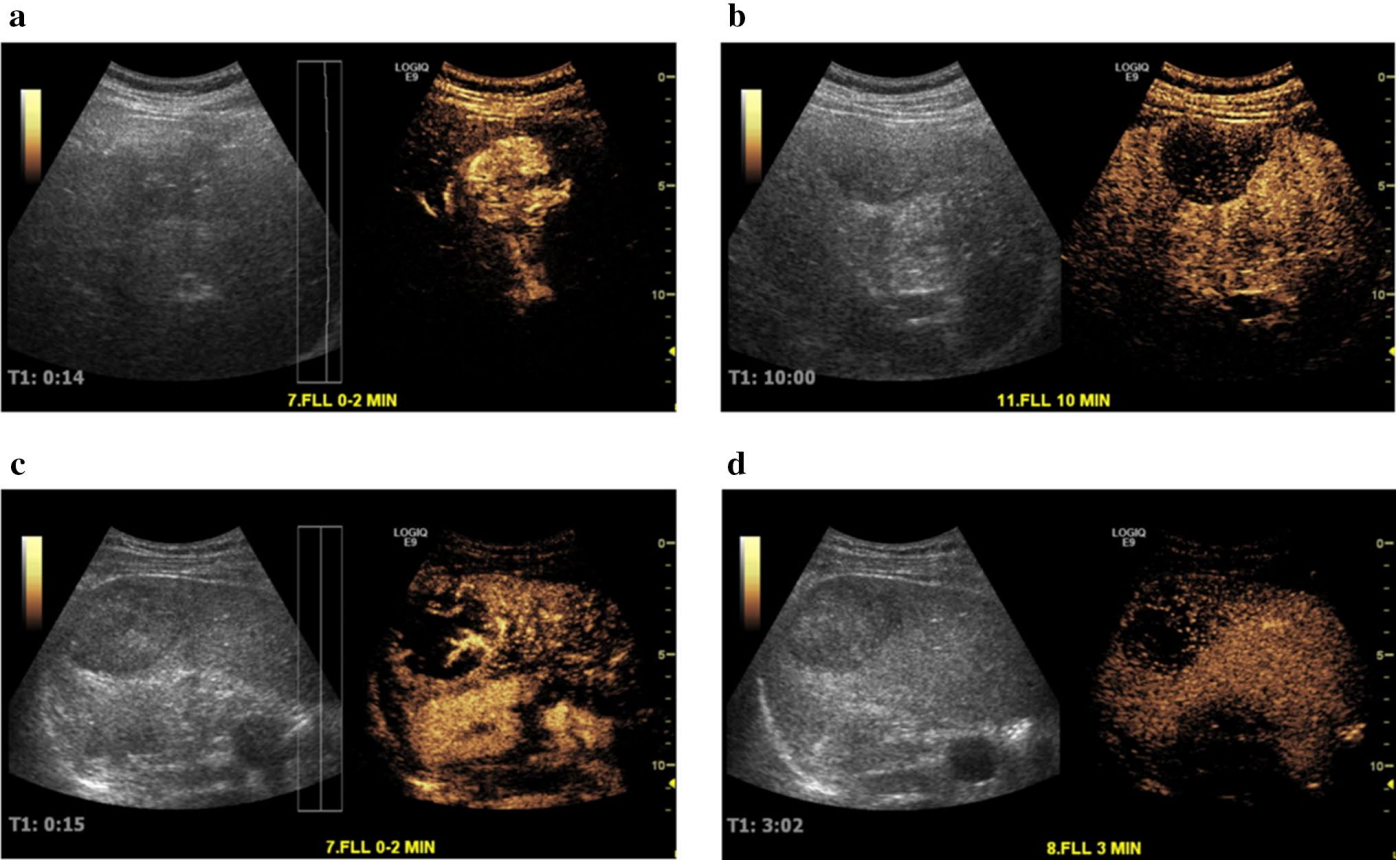
Contrast side-by-side images for a 46-year-old male and a 37-year-old male with hepatocellular carcinoma (HCC) **a**, **b** during the vascular phase (14 s) and the Kupffer phase (10 min) post-Sonazoid administration, and **c**, **d** during the vascular phase (15 s) and the late phase (3 min) post SonoVue **c**, **d** administration, respectively. Kupffer phase imaging with Sonazoid can provide a prolonged and more flexible post-injection imaging window for liver lesion diagnosis

**Fig. 4:**
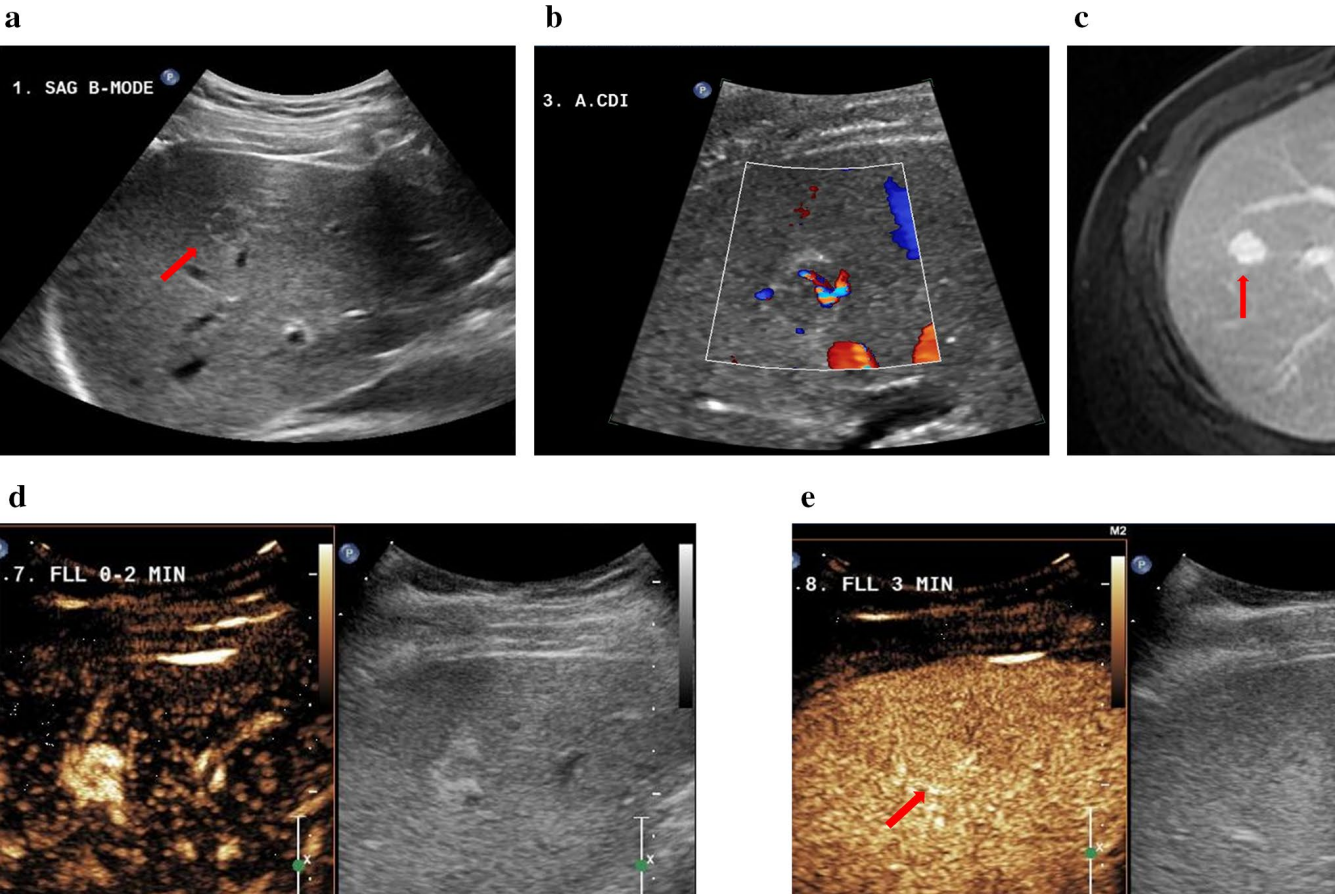
34-year-old male with focal nodular hyperplasia (FNH). **a** Pre-contrast harmonic B-mode showing the lesion. **b** In Colour Doppler imaging the lesion shows central vascularity and some peripheral vascularity. **c** The hyperintense representation of the lesion in the venous/delayed phase on CE-MRI. Imaging performed post-SonoVue injection. **d** Arterial phase shows well-defined lesion margins and central scarring. **e** FNH homogeneous iso-enhancement indicated in the late phase at 3 min

Different types of FLLs have different patterns of enhancement during vascular phase and late/very late phase imaging. Examples of typical enhancement patterns of HCC and FNH on CEUS and reference diagnosis scans are shown in Figs. 2, 3 and 4.

The accuracy improvement with Sonazoid and SonoVue in correctly diagnosing the target lesions as benign or malignant was calculated for each reader and reported in Table 3. For all 3 blinded readers, the 95% upper confidence limit (UCL) for the difference in accuracy improvement between the two groups was within the pre-defined non-inferiority margin of 20%. The *p*-values showed the significance for the non-inferiority tests.

**Table 3:** Comparison of accuracy improvement in diagnosis of target lesions as benign or malignant (post-contrast versus pre-contrast)

Reader	Treatment	Total	Accuracy^a^			Accuracy Improvement (%)	*p*-value^c^
			Improved	Same	Worsened		
1	SonoVue	157	38	100	19	24.2	
	Sonazoid	160	29	114	17	18.1	
	Difference (95% CI^b^)				6.1 ( − 5.0, 17.2)		0.0013
2	SonoVue	158	23	119	16	14.6	
	Sonazoid	163	36	107	20	22.1	
	Difference (95% CI^b^)				− 7.5 (− 18.4, 3.5)		< .0001
3	SonoVue	162	27	115	20	16.7	
	Sonazoid	165	28	121	16	17.0	
	Difference (95% CI^b^)				− 0.3 (− 11.3, 10.7)		< .0001

^a^Post-contrast versus pre-contrast diagnosis when compared with the reference diagnosis

^b^Exact binomial 95% confidence interval

^c^*p*-values based on the Farrington-Manning score test for noninferiority

Table 4 shows the sensitivity, specificity and OA for diagnosing target lesions as benign or malignant. There was no statistically significant difference in the post-contrast OA rates comparing Sonazoid with SonoVue. Within each treatment group, the post-contrast OA was compared to the pre-contrast per reader in Table 4. For both Sonazoid and SonoVue, post-contrast specificity was significantly higher than pre-contrast for all readers (*p* = 0.009, *p* < 0.0001, *p* = 0.001 for reviewers 1,2,3 for Sonazoid; *p* = 0.001, p = 0.03 for reviewer 1 and 2 for SonoVue), except Reader 3 in the SonoVue group (*p* = 0.12). There is a trend for a higher specificity with Sonazoid in comparison with SonoVue but this did not reach significance. Pre-contrast sensitivity was generally slightly higher than post-contrast, but the differences were not statistically significant except for Reader 2 (*p* = 0.049) for Sonazoid group. A similar trend was observed for OA, but with significance achieved for only 1 reader in each group (*p* = 0.03 for Reader 2 for Sonazoid; *p* = 0.01 for Reader 1 for SonoVue). As this study was not powered for this comparison, the significance was not observed universally across readers.

**Table 4:** Summary of sensitivity, specificity and overall agreement (OA) in diagnosis of target lesions (pre- and post-contrast to reference diagnosis): efficacy population

Reader	Time point	Sonazoid group			SonoVue group			*p*-value^a^
		Sensitivity %	Specificity %	OA %	Sensitivity %	Specificity %	OA %	
1	Pre-contrast	77.4	62.4	69.8	80.8	61.5	69.8%	> 0.99
	Post-contrast	75.3	81.0	78.1	83.6	80.0	81.5%	0.49
	*p*-value^b^	0.83	0.009	0.077	> 0.99	0.002	0.012	
2	Pre-contrast	86.9	54.1	70.4	90.4	68.8	78.1%	0.14
	Post-contrast	75.9	85.0	80.4	86.6	82.4	84.2%	0.39
	*p*-value^b^	0.049	< 0.0001	0.033	0.25	0.03	0.26	
3	Pre-contrast	85.7	58.8	72.2	86.3	64.6	74.0%	0.81
	Post-contrast	79.8	80.2	80.0	84.1	74.2	78.4%	0.79
	*p*-value^b^	0.23	0.001	0.07	0.59	0.12	0.31	

^a^*p*-values comparing overall agreement between Sonazoid and SonoVue groups were obtained from the two-sided Fisher’s exact test

^b^*p*-values comparing pre-contrast and post-contrast sensitivity, specificity and overall agreement were obtained from McNemar’s test

**Fig. 5:**
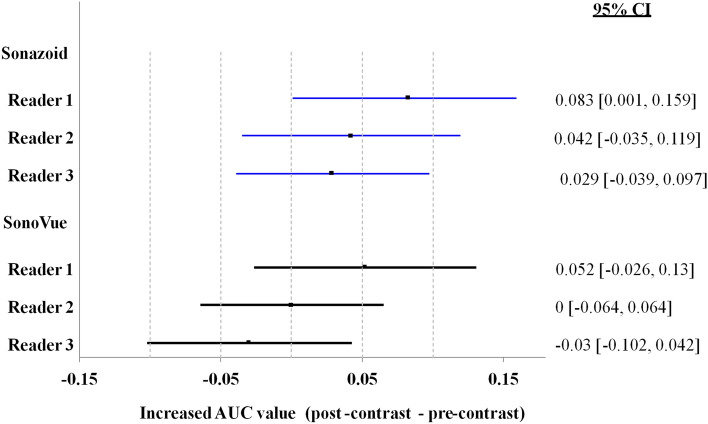
Summary of the area under the curve (AUC) in diagnosis of target lesions (post-contrast–pre-contrast): Efficacy Population

The increased AUC in diagnosing target lesions with the use of contrast agentis shown in Fig. 5. It was noted that the increased AUC was numerically greater in the Sonazoid group than in the SonoVue group across all readers. However, no statistical significance is observed based on Chi-square *p*-values as this study is not powered for this comparison.

The intra-reader variability for diagnostic agreement was also calculated. For all 3 readers, the pre- and post-contrast intra-reader agreement rates were above 80% in both Sonazoid and SonoVue groups. Kappa values indicate moderate to excellent reliability of the 3 readers (Ranged 0.57–1.00 for Sonazoid and 0.53–1.00 for SonoVue). The 3-way congruent intra-reader agreement of the pre- and post-contrast and the reference diagnosis was also assessed using overall agreement in % (ranged from 80 to 100% for Sonazoid and 60% to 87% for SonoVue) and the weighted kappa (1.00, 0.89 and 0.40 for the 3 readers for Sonazoid and 0.79, 0.52 and 0.13 for SonoVue). Based on analysis of the re-read dataset, the result suggested a higher agreement ratein the Sonazoid group.

All 3 readers’ level of diagnostic confidence improved with the post-contrast images as shown in Fig. 5. The level of diagnostic confidence in most of the pre-contrast diagnoses (66.9%, 52.7% and 52.7% for 3 readers in Sonazoid group; 72.2%, 46.7% and 53.30% for SonoVue group) was “probable”, suggesting the reader would have more confidence if another diagnostic imaging test such as CE-MRI or CE-CT was performed. The proportion of cases scored as “definite” (i.e., the reader was sufficiently confident in his or her diagnosis that another diagnostic imaging test such as CE-MRI or CE-CT was unnecessary) considerably increased (by 29.7%, 62.3% and 56.3% for Sonazoid; by 27.9%, 58.1% and 50.9% for SonoVue) for all 3 readers with post-contrast images compared with pre-contrast images.

### Safety results

Safety results are shown in Table 5. Both Sonazoid and SonoVue were well-tolerated. Treatment-emergent AEs (TEAEs) were recorded for 55 (13.0%) of patients: 28 (12.8%) in the Sonazoid group and 27 (13.1%) in the SonoVue group. The majority of these patients (85.4%) experienced TEAEs that were mild in intensity. Thirteen patients (3.1%) experienced TEAEs for which there was a reasonable possibility that the study contrast agent caused the event, including 8 (3.7%) patients in the Sonazoid group and 5 (2.4%) patients in the SonoVue group.

**Table 5:** Overall summary of treatment-emergent adverse events

Category	Sonazoid (*N* = 218) *N* (%)	SonoVue (*N* = 206) *n* (%)	Overall (*N* = 424) *n* (%)
Subjects with any TEAE	28 (12.8)	27 (13.1)	55 (13.0)
Subjects with any SAE	0	0	0
Subjects with any TEAE by intensity			
Mild	24 (11.0)	23 (11.2)	47 (11.1)
Moderate	2 (0.9)	2 (1.0)	4 (0.9)
Severe	2 (0.9)	2 (1.0)	4 (0.9)
Subjects with any TEAD related to IMP	8 (3.7)	5 (2.4)	13 (3.1)
Subjects withdraws due to any AE	0	1 (0.5)	1 (0.2)
Death	0	0	0

A subject who experienced more than 1 occurrence of an AE was counted once for that AE, at the most severe intensity. A subject with multiple AEs within the same category was counted once for that category. TEAE is Treatment-Emergent Adverse Effects. IMP is Investigational Medicinal products

The majority of TEAEs were mild changes in blood pressure (0.7% overall) or heart rate (0.2% overall) with no clinical symptoms and the investigators did not link them with administration of the contrast agent. Abdominal pain was reported by 6 patients in each group (2.8% Sonazoid, 2.9% SonoVue) and for 5 out of 6 cases not related to contrast administration. One patient had a mild TEAE of contusion at the injection site 4 h after the injection of SonoVue.

## Discussion

As a new ultrasound contrast agent, Sonazoid has been applied more and more widely since it was listed on the market. It can not only be used for the differential diagnosis of liver and other organ mass or space-occupying lesion, but also accurately guide the treatment of tumor thermal ablation [3, 22–25].

To the best of our knowledge, this is the first multicenter prospective study comparing two UCAs, in two groups of patients presenting similar characteristics in terms of demography and focal liver lesions. The study showed non-inferior efficacy of Sonazoid compared to SonoVue for characterizing FLLs as benign or malignant, based on a strict blinded review performed by 3 independent readers. As it has been demonstrated by previous studies [3, 26, 27], the agreement of target FLLs diagnoses comparing with the reference diagnosis was higher after contrast injection than using conventional ultrasound imaging for both agents.

It was shown in our study that Sonazoid and SonoVue both had high sensitivity and specificity in distinguishing liver malignancies from benign lesions. Real-time CEUS offers typical enhancement pattern of HCC and metastatic lesions. The former is abundant in tumor vessels which appears as irregular branching image from periphery penetrating the center, followed by tumor hyperenhancement in the early arterial phase and washout in the delayed phase, while the latter exhibit rim-like enhancement in the marginal of nodule with complete kupffer defects in the post-vascular phase, just like “black hole” [28]. A large proportion of false-negative metastatic nodules are the results of atypical enhancement pattern which may be caused by the different primary cancers [29, 30]. Hemangioma also has typical enhancement pattern—peripheral globe-like hyperenhancement followed by gradually centripetal perfusion. For some small hemangiomas, they may be depicted as the whole hyperenhancement which lasted till kupffer phase without contrast agent washout. In one hospital's enrolled cases of this multicenter study, 2 nodules of hemangioma, located in the near field of the image, were misidentified as malignancy, which might be caused by the breaking of bubbles [3].

In terms of diagnostic accuracy, the accuracy rate based on pre-contrast images in this study (74% for SonoVue and 81% for Sonazoid) is about the same as that was reported for previous SonoVue studies (85.8%%) [31–33]. And the accuracy improvements seen in this study for Sonazoid are in line with that was reported by Moriyasu and Itoh (86% pre-contrast and 97% post-contrast in 196 patients) [34]. Maintaining a high accuracy rate may be explained by the site experience with ultrasound, the fact that targeted lesions were to be selected on the basis of a clear delineation, the significant improvement in ultrasound imaging technology since the SonoVue studies were performed, and the ways in which image datasets were acquired, handled and read. Note that the vigorous operator training which mandated training cases to be performed (70/424 patients) and the strict image quality control in this study may have contributed significantly to the higher accuracy rate of unenhanced ultrasound imaging. Another important point to note is that for most of the patients, the diagnosis was already known at the time of the enrollment, and the main criterion for the enrollment was the good visibility of the lesion with conventional ultrasound. However, this did not influence the CEUS results since they were based on a blinded image read with no access to the clinical data or medical history. Additionally, a higher accuracy improvement may have been achieved if both the pre- and post-contrast images had been read together as in clinical practice, instead of being read in separate groups.

While the improvements in accuracy were relatively moderate for both UCAs, there was an observable post-contrast improvement in all readers’ diagnostic confidence level. The level of confidence in the majority of the pre-contrast diagnoses was “probable” whereas the proportion of cases scored as “definite” post-contrast increased considerably for all 3 readers. This result underlines an important potential benefit of CEUS over unenhanced imaging in allowing practitioners to reach a confident diagnosis without having to refer patients for additional imaging exams such as MRI or CT.

One limitation of the study is that the reference diagnosis in most cases (318/338) was based on CE-CT or CE-MRI imaging in combination with previous medical history, and the diagnosis of only a small proportion of patients (20/338) was based on biopsy/histopathology. A preferred follow-up examination should have been performed to confirm the reference diagnosis but in the same time, the clinical context and local incidence of primary liver lesions was indicative of the retained diagnosis. Another limitation is that each patient was not exposed to the two agents meaning that their performances could not be compared in an identical group but in similar representative groups of patients. In addition, although we compared and analyzed the differences in the diagnosis of benign and malignant in each patient with unenhanced ultrasound images, contrast-enhanced and enhanced MRI/ CT in this manuscript as shown in Table 3. This made the results seem unclear and confusing. Because our study only conducted non-inferiority test, in the following articles, we will further analyze the data, conduct the effectiveness test and compare each reader's performance individually to the gold standard, and how the CEUS improved each reader's individual performance as compared to non-contrast US in special diagnosis(such as HCC, malignant lesions or hemangioma, etc.).

The relatively similar performances in terms of sensitivity and OA for Sonazoid and SonoVue in the present study despite the use of vascular phase only for SonoVue and vascular phase and Kupffer phase for Sonazoid might be explained by the following factors. The selection of lesions may have influenced these results in the sense that the vascular phase was clear enough for a full characterization of the lesions. However, we could highlight the trend for a greater specificity of Sonazoid which could indicate that subtle details may have helped the readers assess more completely the lesions. Sonazoid can provide clearer and finer vascular images in early vascular phase, and well-differentiated HCC and high-grade dysplastic nodules often showed an increasing of the avascular areas. It has also been confirmed in other literature [35]. The post-vascular phase has been reported as a valuable adjunct for the diagnosis of HCC by Kudo et al. [26, 36], Wu M et al. [37] and integrated in the Japanese guidelines for HCC diagnosis by the Liver Cancer Study Group of Japan [36]. It should be noted that these results have been obtained in Asian patients, and the translation to western countries should require additional study but the diagnostic improvement comparing pre- to post-contrast imaging reported for both agents would be similar [12].

In conclusion, the data collected in the present study confirmed the diagnostic improvement provided by CEUS over unenhanced ultrasound examinations despite the good performances achieved by the latter. Sonazoid and SonoVue perform similarly in terms of efficacy in diagnosing FLLs as benign or malignant. The differences in physico-chemical characteristics did not seem to influence notably the efficacy of focal liver lesion characterization in the study population. This first multicenter prospective comparative study of two UCAs already approved in several countries showed that, on top of a diagnostic improvement with a better confidence, the diagnostic results provided by CEUS are reliable and independent from the injected UCA, while along-lasting enhancement may add some clinical value.

## Footnotes

1 Trademark of GE Healthcare Ltd., hereafter referred to as Sonazoid.

2 Registered Trademark of Bracco Corporate, hereafter referred to as SonoVue.
